# Regional Expression Profiles of Artemin Protein and GFRα3 mRNA During Postnatal Brain Development in Rats

**DOI:** 10.3390/biology15201855

**Published:** 2026-10-15

**Authors:** Deniz Demirtaş, Burak Gülcen, Hasan Çalışkan

**Affiliations:** 1 Department of Anatomy, Faculty of Medicine, Zonguldak Bülent Ecevit University, 67630 Zonguldak, Türkiye; 2 Department of Anatomy, Faculty of Medicine, Balıkesir University, 10145 Balıkesir, Türkiye; burakgulcen@balikesir.edu.tr; 3 Department of Physiology, Faculty of Medicine, Balıkesir University, 10145 Balıkesir, Türkiye; hasan.caliskan@balikesir.edu.tr

**Keywords:** Artemin, cerebellum, GFRα3, neurodevelopment, postnatal brain development

## Abstract

Artemin, a member of the glial cell line-derived neurotrophic factor family, plays important roles in neuronal processes through GDNF family receptor alpha 3 (GFRα3)-mediated signaling. However, the expression patterns of Artemin and GFRα3 during normal postnatal brain development remain poorly characterized. This study characterized regional Artemin protein and *Gfra3* messenger RNA (mRNA) expression profiles in the rat brain at distinct stages of postnatal development. Male Wistar rats aged 9, 41, and 70 days (*n* = 10/group) were examined. Artemin levels were quantified in the prefrontal cortex, striatum, and cerebellum using enzyme-linked immunosorbent assay (ELISA), whereas *Gfra3* mRNA expression was determined by reverse transcription quantitative polymerase chain reaction (RT-qPCR). Regional and age-related differences were evaluated statistically. No significant age-related differences in Artemin levels were observed within any brain region. However, cerebellar Artemin levels were significantly higher than those in the prefrontal cortex and striatum across all age groups. Age-related changes in *Gfra3* mRNA expression were detected only in the cerebellum, where expression was significantly higher in 41-day-old rats than in both 9- and 70-day-old rats and was also significantly higher in 70-day-old rats than in 9-day-old rats. Regional differences in *Gfra3* mRNA expression were detected only in 41-day-old rats, with significantly higher expression in the cerebellum than in the prefrontal cortex and striatum. These findings provide, to our knowledge, the first comprehensive characterization of Artemin protein and *Gfra3* mRNA expression at distinct stages of normal postnatal brain development and highlight the cerebellum as a region characterized by consistently higher Artemin levels and developmentally variable *Gfra3* mRNA expression, supporting further investigation of Artemin–GFRα3 signaling in this region during postnatal brain development.

## Simple Summary

The brain continues to develop after birth through a series of coordinated biological processes. Understanding the molecules involved in these processes is important for understanding how the brain normally matures. Artemin is a naturally occurring protein that can support the growth and survival of nerve cells by acting through receptors on these cells. However, little is known about how Artemin and its primary receptor, GDNF family receptor alpha 3 (GFRα3), are expressed in the normally developing brain. In this study, we examined Artemin protein levels and *Gfra3* messenger RNA (mRNA) expression in three rat brain regions at different postnatal stages. We found that Artemin levels remained relatively stable with age, whereas *Gfra3* mRNA expression showed age-related changes only in the cerebellum. Regional differences in *Gfra3* mRNA expression were observed only during adolescence, when expression was higher in the cerebellum than in the other brain regions examined. Notably, the cerebellum consistently contained higher levels of Artemin than the other brain regions examined. Together, these findings highlight the cerebellum as a region showing both consistently higher Artemin levels and age-related variation in *Gfra3* mRNA expression and support further investigation of Artemin–GFRα3 signaling in this region during postnatal brain development.

## 1. Introduction

Postnatal brain development is a dynamic process that extends from birth to adulthood [1]. During this period, the central nervous system (CNS) undergoes structural and functional changes, including neurite outgrowth, synaptogenesis, synaptic pruning, and myelination. These processes establish and refine neural circuits [2,3]. The developing brain is particularly vulnerable to adverse environmental and pharmacological exposures, including neurotoxic agents and anesthetics, which may disrupt normal developmental processes and result in persistent neurodevelopmental consequences [4,5]. Consequently, understanding the molecular mechanisms that regulate postnatal brain maturation contributes to the characterization of normal neurodevelopment and developmental abnormalities associated with neurological disorders.

Neurotrophic factors promote neuronal growth, differentiation, survival, and neurite outgrowth in both the central and peripheral nervous systems [6]. These factors also contribute to neural circuit formation and plasticity [7]. Neurotrophic factors include three families of growth factors: neurotrophins, glial cell line-derived neurotrophic factor (GDNF) family ligands, and neuroprotective cytokines [8]. The GDNF family ligands comprise GDNF, neurturin, Artemin, and persephin [9]. GDNF family ligands bind to glycosylphosphatidylinositol-anchored GDNF family receptor alpha 1–4 (GFRα1–4) and activate the transmembrane receptor tyrosine kinase rearranged during transfection (RET); this activation initiates intracellular signaling pathways involved in neuronal development, survival, and functional maturation [10,11].

Artemin preferentially binds to the GDNF family receptor alpha 3 (GFRα3) co-receptor to activate RET, although it can also interact with GFRα1 [12]. RET-mediated signaling involves intracellular pathways including phosphoinositide 3-kinase/protein kinase B (PI3K/AKT) and mitogen-activated protein kinase/extracellular signal-regulated kinase (MAPK/ERK), which are associated with neurotrophic responses such as neuronal survival, neurite growth, and branching [13]. Experimental evidence further indicates that Artemin can promote neuronal survival and differentiation through RET- and neural cell adhesion molecule (NCAM)-associated mechanisms [12]. Previous studies have also demonstrated that Artemin promotes axonal regeneration and functional recovery following neural injury [14]. Furthermore, Artemin has been implicated in sensory neuron development, neuropathic pain, dopaminergic neuronal differentiation, and several neurological and neoplastic disorders [15,16,17,18]. Current knowledge about Artemin has been derived largely from studies examining peripheral nerve regeneration, pathological conditions, or specific neuronal populations [14,15,16,19,20,21]. Although several studies have examined the postnatal expression of other neurotrophic factors and their receptors in the CNS [22,23,24], to our knowledge, the physiological expression profiles of Artemin and GFRα3 during normal postnatal development of the CNS have not been comprehensively characterized.

The prefrontal cortex (PFC), striatum, and cerebellum undergo distinct patterns and timelines of structural and functional maturation and remodeling throughout the postnatal period [25,26,27], which may contribute to regional differences in gene expression and neurotrophic signaling. Therefore, examining the expression profiles of Artemin and GFRα3 within these regions could provide information on the developmental regulation of the Artemin signaling pathway.

Human and rat brain development share substantial similarities in fundamental developmental processes such as neurogenesis, myelination, synaptogenesis, and neuronal and synaptic pruning [28,29]. However, the timing of these stages varies considerably and is regionally heterogeneous across different brain structures [28,29]. Therefore, studying rat brain development at appropriately selected time points may provide information relevant to understanding human brain development.

On this mechanistic and developmental basis, we reasoned that the expression profiles of Artemin and GFRα3 might vary according to developmental stage and brain region. This provided the testable prediction that differences in these expression profiles would be detectable across postnatal stages and/or among brain regions. Under this framework, developmental regulation would be reflected by differences in expression across postnatal stages, whereas regional regulation would be reflected by differences among the three brain regions. Accordingly, the present study aimed to characterize Artemin protein and *Gfra3* messenger RNA (mRNA) expression in the PFC, striatum, and cerebellum of 9-, 41-, and 70-day-old rats and to determine whether these profiles differed across developmental stages and brain regions.

## 2. Materials and Methods

### 2.1. Animals

Three groups were formed from 9-, 41-, and 70-day-old male Wistar albino rats; each group consisted of 10 animals. Sample size was determined using G*Power during the original study design; however, the original G*Power analysis output is not available for retrospective verification. Therefore, a sensitivity analysis was subsequently performed using G*Power version 3.1.9.7 to characterize the statistical sensitivity of the final sample of 30 animals (10 animals per age group). For the repeated-measures within–between interaction, the analysis was specified with three groups, three repeated measurements, α = 0.05, a correlation of 0.50 among repeated measurements, and a nonsphericity correction of 1.0. With these parameters, the available sample provided a statistical power of 80% to detect an effect size of f = 0.27. Animals were assigned to the three study groups according to their chronological age; therefore, random allocation to age groups was not applicable. Within each age group, animal identification numbers (1–10) were assigned randomly, and samples were subsequently processed according to these identification numbers. Nine-day-old rats were selected to represent the neonatal developmental stage, which broadly corresponds to the human infant period [28,29]. We selected 41-day-old rats because they correspond to the adolescent stage [30]. We selected 70-day-old rats because they correspond to the young adult stage [30]. Animals were obtained from the Balıkesir University Experimental Animals Laboratory. Animals were housed in groups of 10 per cage under a 12 h light/dark cycle at a controlled temperature of 22–25 °C and relative humidity of 50 ± 5%, with ad libitum access to standard chow and tap water. No additional environmental enrichment was provided. Predefined humane endpoints included loss of more than 20% of body weight, inability to walk normally, inability to obtain food or water adequately, and markedly reduced responsiveness to stimuli. No animal met these criteria, and no animals were excluded from the study. Ethics committee approval for the present study was obtained from the Balıkesir University Experimental Animals Ethics Committee (Approval No: 2024/12-1, Date: 26 December 2024).

### 2.2. Sacrifice and Tissue Collection

Rats were deeply anesthetized by intraperitoneal injection of ketamine (50 mg/kg) and xylazine (10 mg/kg). Body weight and head length measurements were obtained prior to sacrifice, whereas brain weight was measured following sacrifice. Animals were sacrificed by cardiac exsanguination under deep anesthesia. The PFC, striatum, and cerebellum were then rapidly dissected according to the Paxinos and Watson rat brain atlas [31] for subsequent enzyme-linked immunosorbent assay (ELISA) and reverse transcription quantitative polymerase chain reaction (RT-qPCR) analyses. Tissue samples were stored at −80 °C until further analysis. All procedures from animal housing and handling to sacrifice, tissue collection, and storage at −80 °C were performed at the Balıkesir University Experimental Animals Laboratory. ELISA and RT-qPCR analyses were performed at a separate laboratory. The investigators performing the molecular analyses were not informed of the study hypotheses or expected outcomes. Samples were labeled with animal identification numbers, age information, and brain-region abbreviations during the assays. Head length was defined as the distance between the nose and the posterior border of the skull [32,33]. All experimental procedures were conducted in accordance with the Guide for the Care and Use of Laboratory Animals published by the US National Institutes of Health [34].

### 2.3. Artemin Quantification by ELISA

Tissues were homogenized in 140 mmol/L potassium chloride buffer at a 1:9 ratio (0.1 g tissue: 0.9 mL buffer) and then centrifuged at 7000 revolutions per minute (rpm) and 4 °C for 5 min using a centrifuge (Andreas Hettich GmbH & Co. KG, Tuttlingen, Germany). The supernatants were collected and used for Artemin quantification. Artemin levels were quantified using a Rat Artemin ELISA kit (BT LAB, Bioassay Technology Laboratory, Shanghai, China; Cat. No. E3432Ra) according to the manufacturer’s instructions. According to the manufacturer’s instructions, the assay has a standard curve range of 18.75–1200 ng/L, an analytical sensitivity of 10.37 ng/L, an intra-assay coefficient of variation of <8%, and an inter-assay coefficient of variation of <10%. Tissue homogenates are included among the intended sample types. Absorbance was measured at 450 nm using a microplate reader (ELx800; BioTek Instruments, Inc., Winooski, VT, USA). Samples were analyzed as single measurements without technical replicates. The manufacturer’s tissue-sample preparation procedure specifies a fixed 1:9 tissue weight-to-buffer volume ratio and does not include measurement of total protein concentration or normalization of Artemin concentrations to total protein. Artemin concentrations in the present study were not normalized to total protein concentration.

### 2.4. Quantitative Real-Time Polymerase Chain Reaction

Approximately 50–60 mg of tissue was combined with 0.6 mL of lysis buffer and zirconium beads and homogenized using a Bioprep-6 Homogenizer (Allsheng, Hangzhou, China) at 3200 rpm for 30 s, followed by a 45-s interval and a second 30-s homogenization at 3200 rpm. The homogenate was then centrifuged at 7000 rpm for 5 min at 4 °C, and the resulting supernatant was used for total ribonucleic acid (RNA) isolation. Subsequently, 0.6 mL of 70% ethanol was added to the supernatant, followed by loading onto the RNA column, sequential washing with Wash Buffer I and Wash Buffer II, and final elution of purified RNA in 50 µL of ribonuclease (RNase)-free water. Total RNA was isolated using the PureLink RNA Mini Kit (Invitrogen, Carlsbad, CA, USA; Cat. No. 12183018A) according to the manufacturer’s protocol. RNA concentration was determined using a Qubit 4 Fluorometer (Invitrogen, Thermo Fisher Scientific, Waltham, MA, USA) together with the Qubit RNA BR Assay Kit (Invitrogen, Carlsbad, CA, USA; Cat. No. Q10210). RNA integrity was not independently assessed using the RNA integrity number (RIN), Bioanalyzer, or gel electrophoresis.

Complementary DNA (cDNA) was synthesized from 0.5 μg of total RNA using the Otto cDNA Synthesis Kit (Otto Scientific, Ankara, Türkiye; Cat. No. OttoP1152) in a total reaction volume of 20 μL according to the manufacturer’s protocol. Reverse transcription was performed at 42 °C for 60 min, followed by enzyme inactivation at 80 °C for 10 min, and the resulting cDNA was stored at −20 °C until analysis. RT-qPCR was performed using 2× SYBR Mix (Otto Scientific, Ankara, Türkiye; Cat. No. OttoP1854) on an Insta Q96 Plus Real-Time Polymerase Chain Reaction (PCR) System (HiMedia, Mumbai, India). Each 20-μL reaction contained 10 μL of 2× SYBR Mix, 0.4 μL each of forward and reverse primers, and 2 μL of cDNA, with nuclease-free water added to a final volume of 20 μL. The amplification protocol consisted of an initial denaturation at 95 °C for 5 min, followed by 40 cycles of 95 °C for 10 s, 60 °C for 15 s, and 72 °C for 20 s, with a final extension at 72 °C for 2 min.

Gene-specific primers for *Gfra3* and the reference gene *Actb* were used (Table 1). The *Gfra3* and *Actb* primers were designed using National Center for Biotechnology Information (NCBI) Primer-BLAST (https://www.ncbi.nlm.nih.gov/tools/primer-blast/, accessed on 21 September 2026) against *Rattus norvegicus* sequences and synthesized by Oligomer. The *Gfra3* primers generated a 131-base-pair (bp) amplicon corresponding to accession NM_053398.1, whereas the *Actb* primers generated an 89-bp amplicon corresponding to accession NM_031144.3. These primer sets had previously been established and used in the laboratory. Experiment-specific serial-dilution standard-curve reports were not retained in the available records; therefore, experiment-specific amplification-efficiency and R^2^ values are not available. Melt-curve analysis was not performed specifically for the present experiment, and corresponding experiment-specific melt-curve data are therefore not available.

No-template controls (NTC) and no-reverse-transcription (no-RT) controls were included on the quantitative PCR (qPCR) plates to monitor reagent contamination and genomic DNA contamination, respectively. No amplification was detected in these control wells (cycle threshold [Ct] undetermined). However, the original well-map/output files documenting these controls were not retained in the summarized dataset available for the present revision.

For each animal and brain region, two separate tissue portions were independently processed for RNA extraction, cDNA synthesis, and qPCR analysis. The mean of the two measurements was used as the expression value for that animal and brain region. These measurements therefore represent technical replication of the molecular workflow rather than additional biological replicates. Experiment-specific intra- and inter-assay coefficient of variation (CV) values for RT-qPCR are not available from the retained experimental records. Relative *Gfra3* mRNA expression was calculated using the 2^−ΔΔCt^ method following normalization to *Actb* expression.

### 2.5. Statistical Analysis

The principal molecular outcome measures of the study were Artemin protein levels and *Gfra3* mRNA expression across the three brain regions and developmental ages examined. Morphometric measurements were considered secondary outcome measures. Statistical analyses and graph generation were performed using GraphPad Prism version 10.5 (GraphPad Software, Boston, MA, USA). Data are presented as mean ± standard error of the mean (SEM) for morphometric variables in Table 2, whereas Artemin levels and *Gfra3* mRNA expression are presented as individual observations with the median and interquartile range (IQR) in the figures.

For the analyses of Artemin levels and *Gfra3* mRNA expression, age (9, 41, and 70 days) was treated as a between-subject factor and brain region (prefrontal cortex, striatum, and cerebellum) as a within-subject factor, because the three brain regions were obtained from the same animal, whereas different animals were used at each age. Artemin levels and relative *Gfra3* mRNA expression values (2^−ΔΔCt^) were log_10_-transformed prior to statistical analysis because residual diagnostics of the models fitted to the untransformed data indicated deviations from normality and homoscedasticity. Log_10_ transformation substantially improved the residual distributions and variance homogeneity. Artemin data were analyzed using two-way repeated-measures analysis of variance (ANOVA), whereas *Gfra3* mRNA expression data were analyzed using a mixed-effects model fitted by restricted maximum likelihood (REML) because four qPCR measurements could not be obtained due to insufficient sample material (one striatal and one cerebellar measurement at postnatal day 41 [P41], and one striatal and one cerebellar measurement at postnatal day 70 [P70]). These values were treated as missing and were not imputed. In both analyses, the Geisser–Greenhouse correction was applied without assuming sphericity. Tukey’s multiple-comparisons test was used for pairwise comparisons among ages within each brain region and among brain regions within each age group. Partial η^2^ was reported as the effect-size measure for the main effects of age and brain region and for the age × brain region interaction, and 95% confidence intervals were reported for the estimated mean differences in post hoc pairwise comparisons. Model assumptions were evaluated by visual inspection of quantile–quantile plots, residual-versus-predicted plots, and homoscedasticity plots.

To assess the potential influence of reference-gene variation, raw *Actb* Ct values were additionally analyzed across developmental ages and brain regions using two-way repeated-measures ANOVA with the Geisser–Greenhouse correction. As a sensitivity analysis independent of *Actb* normalization, raw *Gfra3* Ct values were analyzed using a mixed-effects model fitted by REML because of the four missing measurements described above. Tukey’s multiple-comparisons test was used for the corresponding pairwise comparisons. The raw *Gfra3* Ct analysis was interpreted only as a sensitivity analysis and not as an alternative normalized measure of gene expression.

Normality of the morphometric variables (body weight, head length, and brain weight) was assessed using the Shapiro–Wilk test. For each morphometric variable, comparisons among the 9-, 41-, and 70-day-old groups were performed using one-way analysis of variance (ANOVA) followed by Tukey’s multiple-comparisons test when the data were normally distributed. For non-normally distributed morphometric variables, the Kruskal–Wallis test followed by Dunn’s multiple-comparisons test was applied.

For graphical presentation of relative *Gfra3* mRNA expression, 2^−ΔΔCt^ values were calculated using comparison-specific calibrators. For age-dependent presentation, the mean ΔCt value of the 9-day-old group was used as the calibrator within each brain region. For regional presentation, the mean ΔCt value of the PFC was used as the calibrator within each age group. Inferential statistical analyses of *Gfra3* mRNA expression were performed on log_10_-transformed relative expression values, as described above.

## 3. Results

### 3.1. Morphometric Findings

Body weight, brain weight, and head length increased progressively with postnatal age (Table 2). Body weight differed significantly among age groups (Kruskal–Wallis, H(2) = 26.12, *p* < 0.0001), with Dunn’s multiple-comparisons test showing significant differences between 9- and 41-day-old (adjusted *p* = 0.0327), 9- and 70-day-old (adjusted *p* < 0.0001), and 41- and 70-day-old rats (adjusted *p* = 0.0327). Head length also differed significantly among age groups (Kruskal–Wallis, H(2) = 26.10, *p* < 0.0001), with significant differences between 9- and 41-day-old (adjusted *p* = 0.0319), 9- and 70-day-old (adjusted *p* < 0.0001), and 41- and 70-day-old rats (adjusted *p* = 0.0319). Brain weight differed significantly among age groups (one-way ANOVA, F(2, 27) = 486.2, *p* < 0.0001).

### 3.2. Artemin Levels

**Figure 1:**
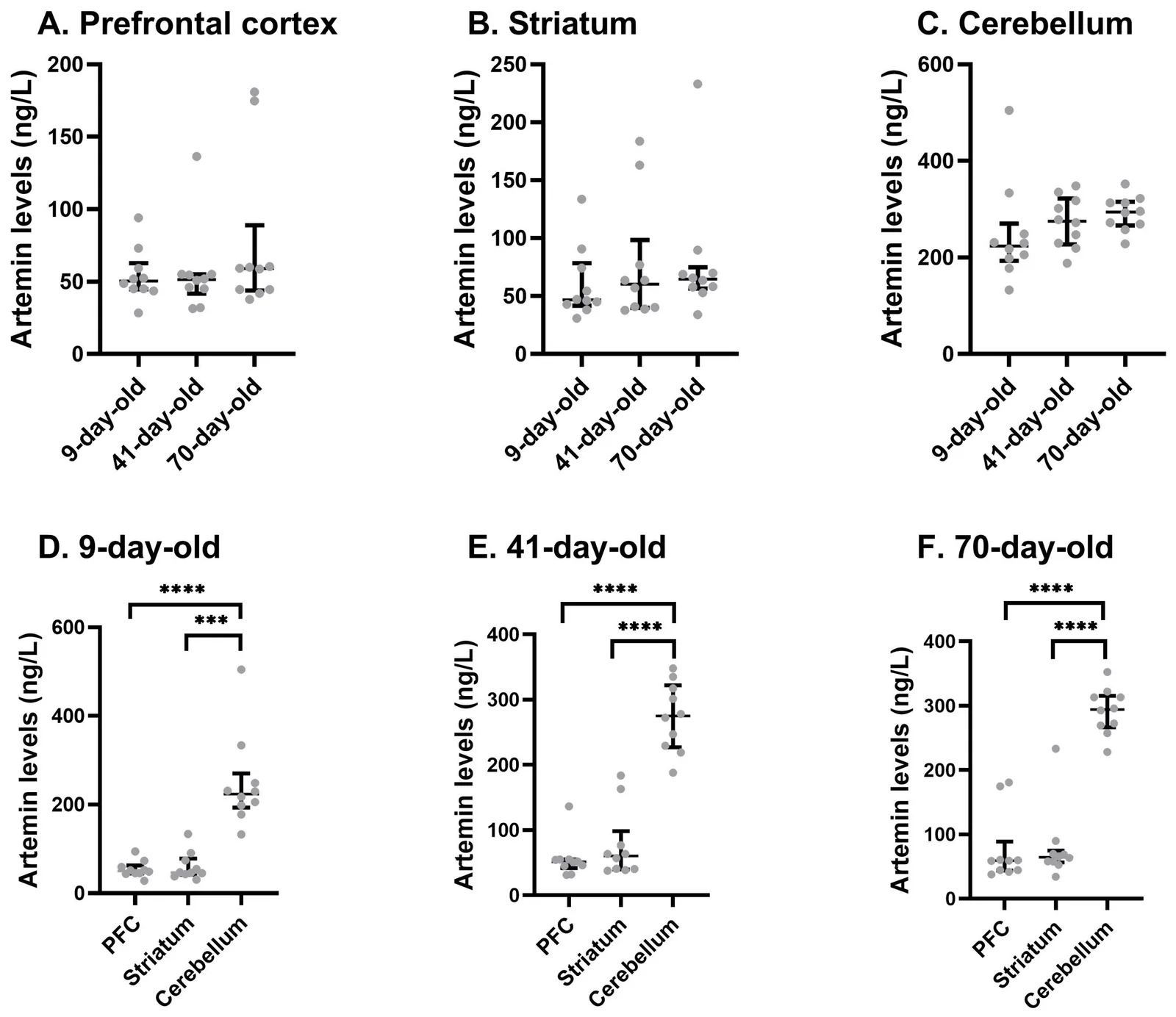
Artemin levels across postnatal ages and brain regions in the rat brain. Artemin levels in the prefrontal cortex (PFC), striatum, and cerebellum of 9-, 41-, and 70-day-old rats are shown. (**A**–**C**) Age-dependent distributions of Artemin levels in the PFC (**A**), striatum (**B**), and cerebellum (**C**). (**D**–**F**) Regional distributions of Artemin levels in 9-day-old (**D**), 41-day-old (**E**), and 70-day-old (**F**) rats. Individual data points represent individual animals (*n* = 10 per age group); horizontal lines and error bars indicate the median and interquartile range (IQR), respectively. Statistical analyses were performed on log_10_-transformed data using two-way repeated-measures analysis of variance (ANOVA), with age as a between-subject factor and brain region as a within-subject factor. The main effect of brain region was significant, *F*(1.824, 49.24) = 125.9, *p* < 0.0001, partial η^2^ = 0.823, whereas the main effect of age, *F*(2, 27) = 2.465, *p* = 0.1039, partial η^2^ = 0.154, and the age × brain region interaction, *F*(4, 54) = 0.1637, *p* = 0.9558, partial η^2^ = 0.012, were not significant. Tukey’s multiple-comparisons test was used for pairwise comparisons. *** adjusted *p* < 0.001; **** adjusted *p* < 0.0001.

Statistical analyses of Artemin levels were performed on log_10_-transformed data. No significant age-dependent differences in Artemin levels were observed within the PFC, striatum, or cerebellum (Figure 1A–C). Consistent with this observation, the main effect of age was not statistically significant, F(2, 27) = 2.465, *p* = 0.1039, partial η^2^ = 0.154, and there was no significant age × brain region interaction, F(4, 54) = 0.1637, *p* = 0.9558, partial η^2^ = 0.012.

In contrast, there was a significant main effect of brain region, F(1.824, 49.24) = 125.9, *p* < 0.0001, partial η^2^ = 0.823. Tukey’s multiple-comparisons tests showed that Artemin levels were significantly higher in the cerebellum than in the PFC and striatum at all three developmental stages (Figure 1D–F). At postnatal day 9 (P9), the mean differences were −0.6534 for PFC vs. cerebellum (95% confidence interval [CI], −0.8850 to −0.4219; adjusted *p* < 0.0001) and −0.6277 for striatum vs. cerebellum (95% CI, −0.8773 to −0.3781; adjusted *p* = 0.0002). At P41, the corresponding mean differences were −0.7199 (95% CI, −0.8619 to −0.5779; adjusted *p* < 0.0001) and −0.6187 (95% CI, −0.8384 to −0.3991; adjusted *p* < 0.0001), respectively. At P70, they were −0.6521 (95% CI, −0.8583 to −0.4458; adjusted *p* < 0.0001) and −0.6211 (95% CI, −0.8303 to −0.4119; adjusted *p* < 0.0001), respectively. Artemin levels in the PFC and striatum did not differ significantly at any age (all adjusted *p* > 0.05).

### 3.3. Gfra3 mRNA Expression

Statistical analyses of relative *Gfra3* mRNA expression were performed on log_10_-transformed 2^−ΔΔCt^ values. The mixed-effects model revealed a significant main effect of age, F(2, 77) = 24.57, *p* < 0.0001, partial η^2^ = 0.390, a significant main effect of brain region, F(1.637, 63.02) = 18.05, *p* < 0.0001, partial η^2^ = 0.319, and a significant brain region × age interaction, F(4, 77) = 6.075, *p* = 0.0003, partial η^2^ = 0.240.

**Figure 2:**
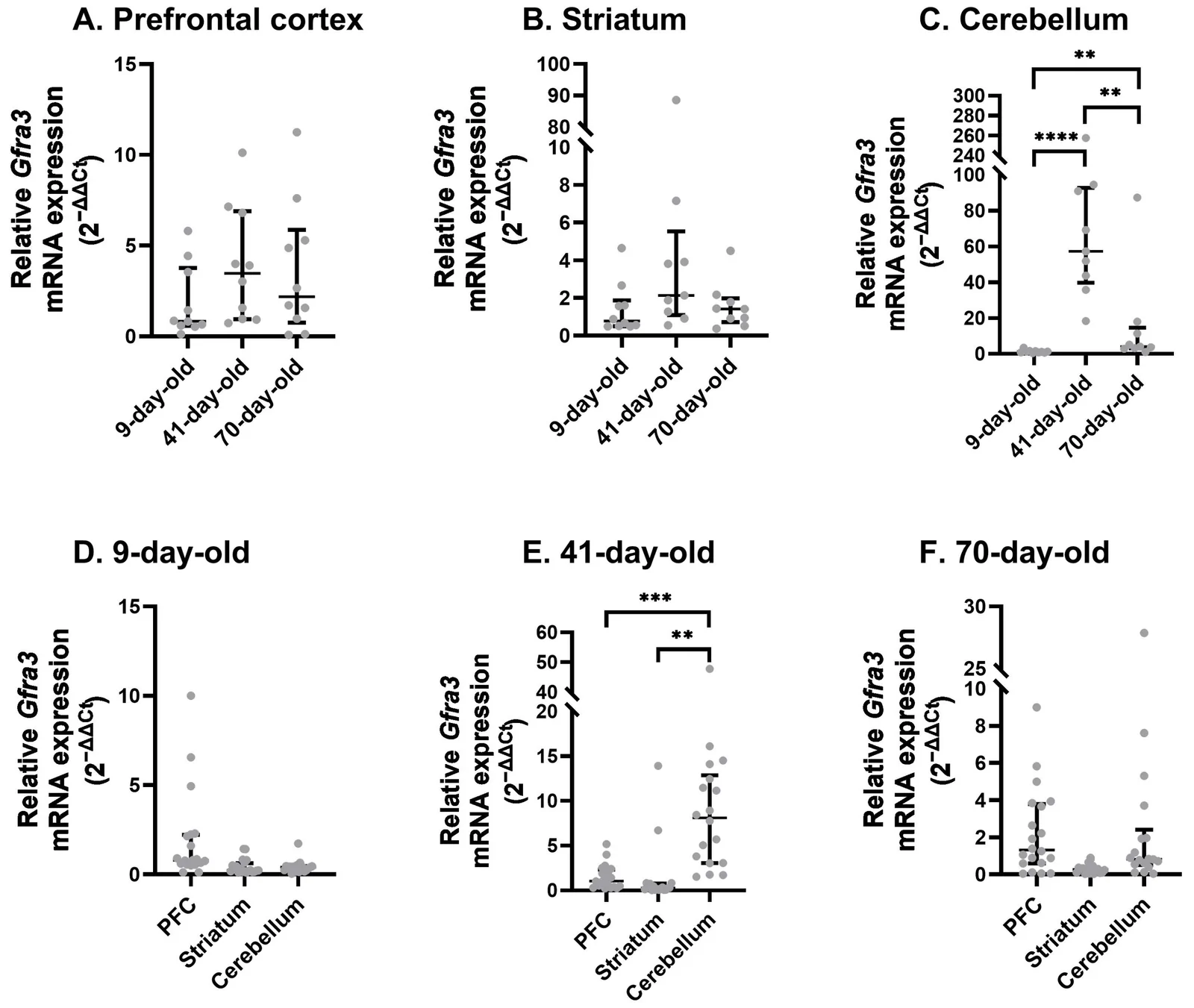
Age- and brain region-dependent distribution of *Gfra3* messenger RNA (mRNA) expression in the rat brain. Relative *Gfra3* mRNA expression in the prefrontal cortex (PFC), striatum, and cerebellum of 9-, 41-, and 70-day-old rats is shown. (**A**–**C**) Age-dependent distributions of *Gfra3* mRNA expression in the PFC (**A**), striatum (**B**), and cerebellum (**C**). (**D**–**F**) Regional distributions of *Gfra3* mRNA expression in 9-day-old (**D**), 41-day-old (**E**), and 70-day-old (**F**) rats. Individual data points represent individual animals; horizontal lines and error bars indicate the median and interquartile range (IQR), respectively. Sample sizes were *n* = 10 for all PFC groups and the postnatal day 9 (P9) striatum and cerebellum groups, and *n* = 9 for the postnatal day 41 (P41) and postnatal day 70 (P70) striatum and cerebellum groups because of four missing quantitative polymerase chain reaction (qPCR) values. For improved visualization of the individual observations, broken Y-axes are used in panels B, C, E, and F. Statistical analyses were performed on log_10_-transformed 2^−ΔΔCt^ values using a mixed-effects model fitted by restricted maximum likelihood (REML), with age as a between-subject factor and brain region as a within-subject factor. The main effects of age, F(2, 77) = 24.57, *p* < 0.0001, partial η^2^ = 0.390, and brain region, F(1.637, 63.02) = 18.05, *p* < 0.0001, partial η^2^ = 0.319, as well as the age × brain region interaction, F(4, 77) = 6.075, *p* = 0.0003, partial η^2^ = 0.240, were significant. Tukey’s multiple-comparisons test was used for pairwise comparisons. ** adjusted *p* < 0.01; *** adjusted *p* < 0.001; **** adjusted *p* < 0.0001.

Age-dependent changes in *Gfra3* mRNA expression were observed only in the cerebellum (Figure 2A–C). Cerebellar *Gfra3* mRNA expression was significantly higher at P41 than at P9 (mean difference = −1.749, 95% CI, −2.092 to −1.406; adjusted *p* < 0.0001) and P70 (mean difference = 0.9999, 95% CI, 0.4339 to 1.566; adjusted *p* = 0.0012), and was also significantly higher at P70 than at P9 (mean difference = −0.7491, 95% CI, −1.294 to −0.2038; adjusted *p* = 0.0090). No significant age-dependent differences were detected in the PFC or striatum (all adjusted *p* > 0.05).

Regional comparisons showed no significant differences in *Gfra3* mRNA expression among the PFC, striatum, and cerebellum at P9 or P70 (all adjusted *p* > 0.05). At P41, however, *Gfra3* mRNA expression was significantly higher in the cerebellum than in the PFC (mean difference = −1.353, 95% CI, −1.843 to −0.8640; adjusted *p* = 0.0001) and striatum (mean difference = −1.304, 95% CI, −2.114 to −0.4930; adjusted *p* = 0.0052), whereas the PFC and striatum did not differ significantly (adjusted *p* = 0.9516) (Figure 2D–F).

## 4. Discussion

### 4.1. Principal Findings

To our knowledge, this study presents the first comprehensive characterization of Artemin levels and *Gfra3* mRNA expression in the PFC, striatum, and cerebellum at distinct stages of normal postnatal brain development in rats. The main findings of this study can be summarized as follows: (i) No significant overall effect of age or age × brain region interaction on Artemin levels was detected, and no significant age-related differences in Artemin levels were observed within any of the three brain regions; (ii) Artemin levels in the cerebellum were consistently higher than those in the PFC and striatum; (iii) Age-related changes in *Gfra3* mRNA expression were detected only in the cerebellum, where expression was significantly higher in 41-day-old rats than in both 9- and 70-day-old rats and was also significantly higher in 70-day-old rats than in 9-day-old rats; and (iv) regional differences in *Gfra3* mRNA expression were detected only in 41-day-old rats, with significantly higher expression in the cerebellum than in both the PFC and striatum.

### 4.2. Discussion of Morphometric Findings

In the present study, the body weight, head length, and brain weight of rats in all three age groups were within the ranges previously reported for comparable developmental stages in the literature [32,35,36,37]. These parameters are influenced by both biological and environmental factors [32,36,38]. Therefore, these findings suggest that the animals included in this study followed a normal developmental trajectory with respect to these morphometric parameters.

### 4.3. Artemin Expression During Postnatal Brain Development

Artemin was detected in the PFC, striatum, and cerebellum at all postnatal stages examined. No significant overall effect of age or age × brain region interaction was detected, and no significant age-related differences were observed within any of the three regions. In contrast, a recent study examining another GDNF family ligand, persephin, demonstrated age-dependent changes in the PFC, striatum, and cerebellum during postnatal development [39]. Age-dependent changes in brain-derived neurotrophic factor (BDNF) levels have also been reported in the frontal cortex during postnatal development [22].

Previous studies have demonstrated the presence of Artemin in the PFC and suggested potential associations with its functional state [21,40,41,42]. In a study by Çalışkan and Koçak, rats subjected to a lipopolysaccharide-induced neuroinflammation model showed reduced Artemin levels in the PFC, and this reduction was associated with depression-like behaviors [40]. In contrast, another study using a similar lipopolysaccharide-induced depression model reported that inflammation increased Artemin levels in the PFC; however, the antidepressant agent administered in that study was also found to increase Artemin levels [41]. Collectively, these findings indicate that Artemin may be associated with PFC function. The present study extends these observations by demonstrating its persistent presence throughout postnatal PFC development. It has been shown that GDNF is associated with the structure and function of the PFC and may play important roles in neurodegenerative and psychiatric disorders such as Parkinson’s disease and depression [43,44,45,46,47,48,49]. Given that Artemin can signal through GFRα3 and can also activate GFRα1 [12], its presence in the PFC raises the possibility of involvement in GDNF-family receptor signaling; however, the functional significance of this finding was not examined in the present study.

Previous studies on Artemin in the striatum have primarily focused on experimental models involving dopaminergic dysfunction and neurodegeneration [40,50,51,52,53]. Decreased Artemin levels in the striatum have been reported in experimental models of depression, and this has been associated with depression-like behaviors [40]. Artemin exerts neurotrophic effects on dopaminergic neurons [16]. It has been shown that Artemin expression increases following selective degeneration of the nigrostriatal dopaminergic pathway [17]. Furthermore, exogenous Artemin administration has attenuated methamphetamine-induced decreases in striatal dopamine levels [51] and enhanced the survival of dopaminergic neurons following 6-hydroxydopamine-induced lesions [52]. In the present study, the persistent presence of Artemin in the striatum throughout postnatal development, together with its known neurotrophic effects on dopaminergic neurons, raises the possibility of a physiological role for Artemin in striatal development and function.

Previous studies investigating Artemin signaling in the cerebellum are limited. Masure et al. reported that GFRα3 is predominantly expressed in the cerebellum and spinal cord within the CNS [54]. Furthermore, Ilieva et al. demonstrated that Artemin promotes neuronal survival and neurite outgrowth in cultured cerebellar granule neurons [12]. Together with these findings, our results indicate that Artemin protein and *Gfra3* mRNA are present in the cerebellum and suggest that Artemin signaling may contribute to cerebellar development and function.

Artemin levels did not differ significantly between the PFC and striatum at any of the three postnatal stages examined. Consistent with our findings, a previous study using a lipopolysaccharide-induced depression model reported no significant differences in Artemin levels between the PFC and striatum in either control or neuroinflammatory conditions [40].

Artemin levels in the cerebellum were significantly higher than those in the PFC and striatum at all postnatal stages examined. Brain regions differ significantly in their cellular composition, developmental trajectories, and maturation timelines [55,56], which may contribute to region-specific patterns of gene expression and neurotrophic signaling. These developmental differences may partially explain the higher Artemin levels observed in the cerebellum in the present study. GDNF is widely expressed in the cerebellum during both development and adulthood and has been implicated in Purkinje cell differentiation and survival [57,58,59]. Moreover, GDNF has been reported to protect the developing cerebellum against ethanol-induced injury and to improve age-related impairments in motor coordination and learning [58,60]. Given that Artemin can also activate GFRα1-mediated signaling [12], the persistent presence and higher levels of Artemin in the cerebellum throughout postnatal development are consistent with the possibility that Artemin contributes to cerebellar development and physiological function. However, an alternative interpretation should also be considered. Because the PFC, striatum, and cerebellum differ markedly in cellular composition and developmental timing [55,56], the regional differences observed in whole-tissue homogenates may partly reflect differences in the abundance or developmental state of Artemin-expressing cell populations rather than differences in Artemin signaling activity per se. Moreover, the present expression data do not establish pathway activation or a causal role for Artemin in cerebellar maturation. Cell-specific localization and functional studies will therefore be required to clarify the biological basis and significance of the higher cerebellar Artemin levels.

### 4.4. Gfra3 mRNA Expression During Postnatal Brain Development

In this study, *Gfra3* mRNA expression was detected in the PFC during all postnatal stages examined; however, no significant age-related difference was observed. Early studies reported highly restricted GFRα3 expression or expression below the detection limits of the methods used in much of the postnatal and adult CNS [61,62,63]. However, subsequent studies have demonstrated GFRα3 expression in several CNS regions using different methodological approaches [54,64,65,66,67]. Notably, Konishi et al. detected GFRα3 at both the mRNA and protein levels in human cortical neurons [64]. Furthermore, repeated acetyl-L-carnitine treatment has been shown to increase Artemin levels in the PFC, and its antidepressant-like effects were abolished by knockdown of either Artemin or GFRα3 [21]. This functional evidence, together with the persistent *Gfra3* mRNA expression observed in the present study, raises the possibility that Artemin–GFRα3 signaling may contribute to PFC function throughout postnatal development.

*Gfra3* mRNA expression was detected in the striatum at all postnatal stages examined, with no significant age-related differences. In contrast to our findings, Baloh et al. reported no detectable GFRα3 expression in the caudate nucleus and putamen of the adult human brain [61]. Our findings therefore provide additional evidence that *Gfra3* is transcriptionally expressed in the striatum throughout postnatal development. Given the reported associations between striatal Artemin levels and behavioral alterations [40] and its neurotrophic effects on midbrain dopaminergic neurons [16], the presence of Gfra3 mRNA in the striatum raises the possibility that Artemin–GFRα3 signaling may be involved in striatal processes, potentially including those related to dopaminergic systems.

*Gfra3* mRNA expression was detected in the cerebellum throughout postnatal development and showed significant age-related variation, with expression being highest in 41-day-old rats. Expression was significantly higher in 41-day-old rats than in both 9- and 70-day-old rats and was also significantly higher in 70-day-old rats than in 9-day-old rats. Masure et al. demonstrated prominent GFRα3 expression in the cerebellum within the CNS using multiple methodological approaches, including RT-qPCR, Northern blot analysis, and in situ hybridization [54]. Although direct information on the developmental regulation of cerebellar *Gfra3* mRNA expression remains limited, Burazin and Gundlach demonstrated distinct temporal expression patterns of other GDNF receptor family members in the developing rat cerebellum [23]. Together, these findings and the age-related pattern observed in the present study are consistent with developmental variation in *Gfra3* mRNA expression in the cerebellum. However, because the cerebellum undergoes substantial changes in cellular composition and maturation during postnatal development, the observed temporal pattern may also reflect changes in the relative abundance or developmental state of GFRα3-expressing cell populations rather than regulation of *Gfra3* mRNA expression within individual cells.

Regional differences in *Gfra3* mRNA expression were detected only in 41-day-old rats. At this developmental stage, *Gfra3* mRNA expression was significantly higher in the cerebellum than in both the PFC and striatum, whereas no significant regional differences were observed in 9- or 70-day-old rats. Previous studies have also demonstrated region-specific expression of GFRα3 within the CNS [54,64,65,66,67]; however, direct developmental comparisons of *Gfra3* mRNA expression among the PFC, striatum, and cerebellum remain limited. The significantly higher *Gfra3* mRNA expression in the cerebellum than in the PFC and striatum in 41-day-old rats is consistent with the prominent cerebellar expression of GFRα3 reported by Masure et al. [54]. Together with the higher cerebellar Artemin levels observed at the same developmental stage, this pattern is compatible with the possibility of greater involvement of Artemin–GFRα3 signaling in the cerebellum during this period. However, an expression-based comparison cannot establish greater signaling activity, and the regional difference may alternatively reflect differences in cellular composition and developmental timing among the three brain regions. Cell-specific localization and functional measurements of pathway activation will therefore be required to distinguish between these interpretations.

### 4.5. Study Limitations

Several limitations should be considered when interpreting the present findings. First, the study examined only three postnatal developmental stages and included only male rats. Consequently, developmental changes occurring between the examined time points and potential sex-related differences in Artemin and GFRα3 expression could not be assessed. In addition, although rats provide a useful model for studying postnatal brain development, species differences in the timing and regional progression of brain maturation should be considered when extrapolating these findings to human neurodevelopment. The original documentation supporting the sample-size determination was not available for retrospective verification. Therefore, a sensitivity analysis was performed using the final sample size and the repeated-measures experimental design, which indicated that the available sample provided 80% power to detect an effect size of *f* = 0.27 for the within–between interaction; consequently, smaller effects may have remained undetected. The study was also restricted to the PFC, striatum, and cerebellum, and the findings should not be generalized to other CNS regions.

Several methodological limitations also apply to Artemin quantification. Artemin concentrations were measured in tissue homogenates prepared using a standardized tissue weight-to-buffer volume ratio but were not normalized to total protein concentration. Samples were analyzed as single measurements without technical replicates, and recovery experiments and brain-region-specific matrix validation were not performed. Although the ELISA manufacturer specifies tissue homogenates among the intended sample types, this does not establish specific assay validation for the PFC, striatum, and cerebellum. Accordingly, the observed regional differences should be interpreted as differences measured under the standardized homogenization conditions used in this study rather than as definitive total-protein-normalized differences in regional Artemin abundance.

The RT-qPCR analyses also have important limitations. *Gfra3* expression was normalized using *Actb* as a single reference gene, whose stability across the developmental stages and brain regions examined was not prospectively validated using multiple candidate reference genes and dedicated stability algorithms. Moreover, *Actb* Ct values showed significant variation under some experimental conditions. Although a sensitivity analysis using raw *Gfra3* Ct values independently of *Actb* normalization showed substantial agreement with the principal regional and developmental patterns observed in the normalized analysis, the results were not fully concordant. Because raw target-gene Ct values do not constitute an alternative normalized measure of gene expression, this analysis should be regarded as a supportive sensitivity analysis rather than an independent validation of the normalized findings. In addition, experiment-specific amplification-efficiency and R^2^ data and melt-curve validation were not available, RNA integrity was not independently assessed using RIN, Bioanalyzer, or gel electrophoresis, and experiment-specific intra- and inter-assay CV values were unavailable. Although the primer sets used in the present study had previously been established and used in the laboratory, this prior laboratory experience does not substitute for experiment-specific validation under the developmental stages and brain regions examined in the present study. These methodological constraints warrant cautious interpretation of the *Gfra3* mRNA findings.

Furthermore, the analyses were based on whole-tissue measurements and therefore do not provide information on the cellular localization of Artemin or GFRα3 within the examined brain regions. The study was designed to characterize physiological expression patterns and did not include functional experiments capable of establishing causal relationships between Artemin–GFRα3 signaling and postnatal brain maturation.

### 4.6. Future Directions

Future studies should investigate additional postnatal developmental stages and include both sexes to better define the temporal dynamics of Artemin and GFRα3 expression and potential sex-related differences. Moreover, extending these analyses to other cortical, subcortical, and brainstem regions would provide a more comprehensive understanding of the regional distribution of Artemin signaling within the CNS. Integrating protein expression analyses, immunohistochemical localization, and functional approaches will also be important for clarifying the physiological roles and underlying mechanisms of the Artemin–GFRα3 signaling pathway during normal brain development. Future studies should also strengthen the methodological assessment of Artemin and GFRα3 expression by incorporating total-protein normalization and appropriate assay validation for Artemin quantification, as well as multiple experimentally validated reference genes for RT-qPCR analyses. Region-specific investigations are also warranted. In particular, future studies should determine the regional and cellular distribution of Artemin and GFRα3 within the cerebellum and investigate the functional significance of Artemin–GFRα3 signaling during cerebellar development.

## 5. Conclusions

To our knowledge, this study provides the first comprehensive characterization of Artemin protein levels and *Gfra3* mRNA expression in the PFC, striatum, and cerebellum at distinct stages of normal postnatal brain development in rats. Artemin protein levels remained relatively stable within each brain region across the postnatal stages examined, whereas *Gfra3* mRNA expression showed age-related variation specifically in the cerebellum, with expression highest in 41-day-old rats. Regional differences in *Gfra3* mRNA expression were also restricted to 41-day-old rats, with higher expression in the cerebellum than in the PFC and striatum. Notably, Artemin levels were consistently higher in the cerebellum than in the other two regions across all developmental stages examined. Together, these findings highlight the cerebellum as a region characterized by consistently higher Artemin levels and developmentally variable *Gfra3* mRNA expression, supporting further investigation of Artemin–GFRα3 signaling in this region during postnatal brain development.

## Figures and Tables

**Table 1:** Forward and reverse primer sequences for reverse transcription quantitative polymerase chain reaction (RT-qPCR).

*GFRα3* *Rattus norvegicus*	Sequence (5′ → 3′)	Product Length	Accession Number
Forward Primer	AGCACAGCAACTCAGGAACA	131	NM_053398.1
Reverse Primer	CGTCCAACTCGTAGTCACCAA		
***Actb*** ***Rattus norvegicus***	**Sequence (5′ → 3′)**		
Forward Primer	TCAACACCCCAGCCATGTAC	89	NM_031144.3
Reverse Primer	TCCGGAGTCCATCACAATGC		

**Table 2:** Morphometric measurements of rats at different postnatal ages (mean ± standard error of the mean [SEM]).

	9-Day-Old Rats (*n* = 10)	41-Day-Old Rats (*n* = 10)	70-Day-Old Rats (*n* = 10)
Body weight (g)	11.10 ± 0.28	135.50 ± 2.22	285.70 ± 6.94
Head length (mm)	24.85 ± 0.18	41.35 ± 0.35	48.55 ± 0.46
Brain weight (g)	0.810 ± 0.024	1.680 ± 0.021	1.906 ± 0.033

## Data Availability

The data supporting this study’s findings are available from the corresponding author upon reasonable request.

## Abbreviations

The following abbreviations are used in this manuscript:

AKT	Protein kinase B
ANOVA	Analysis of variance
BDNF	Brain-derived neurotrophic factor
bp	Base pair
CI	Confidence interval
CNS	Central nervous system
Ct	Cycle threshold
CV	Coefficient of variation
ELISA	Enzyme-linked immunosorbent assay
ERK	Extracellular signal-regulated kinase
GDNF	Glial cell line-derived neurotrophic factor
GFRα1–4	GDNF family receptor alpha 1–4
GFRα3	GDNF family receptor alpha 3
IQR	Interquartile range
MAPK	Mitogen-activated protein kinase
mRNA	Messenger RNA
NCAM	Neural cell adhesion molecule
NCBI	National Center for Biotechnology Information
NTC	No-template control
no-RT	No-reverse-transcription
P9, P41, P70	Postnatal days 9, 41, and 70, respectively
PCR	Polymerase chain reaction
PFC	Prefrontal cortex
PI3K	Phosphoinositide 3-kinase
qPCR	Quantitative polymerase chain reaction
REML	Restricted maximum likelihood
RET	Rearranged during transfection
RIN	RNA integrity number
RNA	Ribonucleic acid
RNase	Ribonuclease
rpm	Revolutions per minute
RT-qPCR	Reverse transcription quantitative polymerase chain reaction
SEM	Standard error of the mean
